# Confirmatory factor analysis of the Dutch Screening Visual Complaints questionnaire in people with multiple sclerosis

**DOI:** 10.1186/s41687-022-00443-0

**Published:** 2022-04-09

**Authors:** Fleur E. van der Feen, Gera A. de Haan, Iris van der Lijn, Anselm B. M. Fuermaier, Thea J. Heersema, Jan F. Meilof, Joost Heutink

**Affiliations:** 1 Department of Clinical and Developmental Neuropsychology, Faculty of Behavioural and Social Sciences, University of Groningen, a.t.t.n. Fleur van der Feen, Grote Kruisstraat 2/1, 9712 TS Groningen, The Netherlands; 2 Centre of Expertise for Blind and Partially Sighted People, Royal Dutch Visio, PO box 1180, 1270 BD Huizen, The Netherlands; 3 Department of Neurology, University of Groningen, University Medical Centre Groningen, PO box, 30001, 9700 RB Groningen, The Netherlands; 4 Department of Neurology, Martini Hospital Groningen, Martini Ziekenhuis, PO box 30033, 9700 RM Groningen, The Netherlands

**Keywords:** Screening Visual Complaints questionnaire, Multiple sclerosis, Visual complaints, Rehabilitation, Factor analysis

## Abstract

**Background:**

Visual complaints among people with multiple sclerosis (pwMS) are common, but often difficult to recognize. The Screening Visual Complaints questionnaire (SVCq) has been developed to screen for visual complaints in people with a neurodegenerative disease, including multiple sclerosis (MS). A previous study performed a factor analysis in a normal population which revealed an acceptable one-factor model, a three-factor model and a five-factor model within the SVCq. To increase the usability of the SVCq in people with MS, the purpose of the current study was to investigate the fit of the three models in a cohort of pwMS.

**Results:**

The confirmatory factor analysis on the SVCq in 493 people with MS showed good fit for all the models. The three-factor model (*diminished visual perception*, *altered visual perception* and *ocular discomfort*) outperformed the one-factor model. The five-factor model outperformed both models, which showed that dividing the first factor (*diminished visual perception)* into three more factors (*function-related*, *luminance-related* and *task-related*) has merit.

**Conclusions:**

All models may be useful in clinical care for pwMS. The one-factor model may give a quick overview of the presence and severity of visual complaints in general. The individual factors, of either the three- or the five factor models, may contribute to a better recognition of the nature of visual complaints in pwMS and may guide further steps in rehabilitation for pwMS with visual complaints.

## Background

People with a neurodegenerative disorder, such as Parkinson’s disease, dementia and multiple sclerosis (MS) frequently deal with visual problems [1–3]. However, since these problems may not be easy to describe and since symptoms that are more eminent, visible, or acute may receive greater emphasis, visual problems and their impact on daily life’s challenges may remain unattended to in clinical settings [4, 5].

A recent study by this author [6] found that the prevalence of visual complaints among people with MS (pwMS) is higher than previously estimated. Up to 90% of pwMS reported to experience some kind of visual complaints in daily life. Complaints regarding light, such as a difficulty adapting to lighter or darker environments, being blinded by bright light or needing more light were especially common, next to for example the feeling to need more time to see something, changes in the visual field, or problems with depth perception. Overall, the nature of the complaints showed a large variability. Furthermore, the study revealed that pwMS with and without a history of optical neuritis (ON) reported similar complaints and the complaints could occur anytime along the disease course.

The study by van der Feen [6] made use of the Screening Visual Complaints questionnaire (SVCq; [7], see Appendix 1 and 2), a questionnaire that has been developed to quickly screen for the presence and nature of visual complaints in people with Parkinson’s disease, dementia and MS. The SVCq assesses subjective visual complaints on a functional level, while previously used questionnaires primarily assess vision related quality of life in daily activities, such as the NEI-VFQ, the NEI-VFQ-25 or MSVQ-7 [8–10]. The SVCq is comprised of 19 items reflecting visual complaints, which could be rated on frequency of occurrence. The SVCq may help direct attention to visual complaints and may support in referring people with visual complaints to fitting care or rehabilitation.

**Fig. 1:**
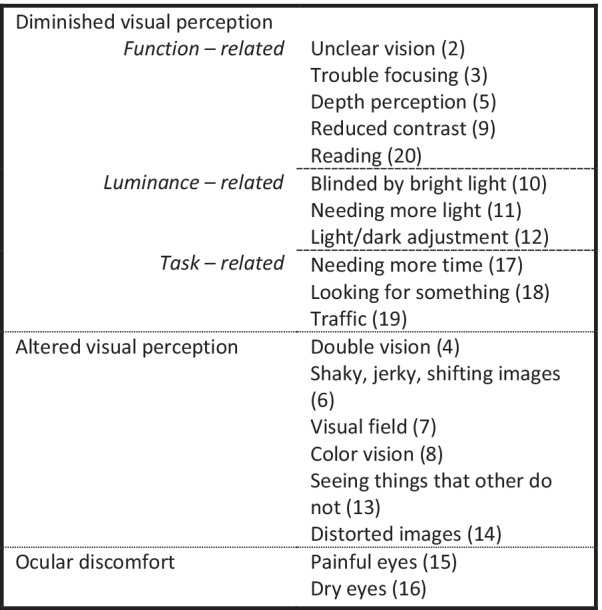
Scales of the SVCq [7] and its items. *The numbers in brackets represent the item number in the SVCq

In a normal Dutch sample (people without MS or other severe neurological, ophthalmological or psychiatric disorders), the SVCq showed satisfactory validity, good internal consistency (α = 0.85), and good test–retest reliability (ICC = 0.82; [7]). In addition, Huizinga [7] performed a factor analysis and proposed a three-factor model of the 19 items, including the factors *diminished visual perception*, *altered visual perception*, and *ocular discomfort*. Along with the proposed three-factor structure, Huizinga [7] found an acceptable fit for the one-factor model (all 19 items comprised in one factor) and a five-factor model, where the *diminished visual perception* factor from the three-factor model was divided into three separate factors, being *function related, luminance related* and *task related* (see Fig. 1 for a summary of the models and their corresponding items).

These promising factor models should however also be investigated in clinical samples, since the SCVq was specifically developed for people with Parkinson’s disease, dementia and MS. Van der Lijn et al. (submitted) investigated the fit of the three models in a representative sample of people with Parkinson’s disease and was able to confirm good fit of all three models.

In order to be able to make optimal use of the SVCq in clinical care for pwMS, we aim to assess the factor structures of Huizinga [7] in a clinical sample of pwMS, using confirmatory factor analysis (CFA).

## Methods

In this study, we made use of the same data set that was used in a study by van der Feen [6].

### Participants

A Dutch cohort of patients regularly (once or twice a year) visiting the MS Centrum Noord Nederland (MSCNN), were invited to complete the SVCq [7]. Since the questionnaire was only available in Dutch at the time of the data collection of this study, only Dutch-speaking patients were invited to complete the questionnaire (SVCq is available in English now; see [7]). Furthermore, the treating neurologist made a decision on clinical basis not to invite people to complete the SVCq when the SVCq would not be of appropriate and additive value to the care of a patient due to too servere disability (cognitively or otherwise). The neurologists estimated that this was the case in 10 to 15 people. In total, we send out 607 questionnaires. Several patients filled out the questionnaire more than once, in which case only the first SCVq was used. From the 607 questionnaires, we received questionnaires back from 507 unique patients, of which 493 patients provided informed consent to use the data for scientific purposes. Demographics and MS-related characteristics of the sample are presented in Table 1.

**Table 1:** Demographics and disease characteristics of the cohort of people with MS

	N	%	M (SD)
Sex (female)	346	70	
Age (y)	493		50.66 (13.17)
Education			
Low	56	11.4	
Intermediate	229	46.5	
High	208	42.2	
Type of MS			
RRMS	237	48.1	
SPMS	175	35.5	
PPMS	43	8.7	
Other	24	4.9	
Unknown	14	2.8	
EDSS			
0–3	204	41.4	
3.5–5.0	130	26.4	
5.5–7.0	119	24.1	
7+	22	4.5	
Unknown	18	3.7	
Time since diagnosis (y)	474		14.01 (10.67)
Unknown	19	3.9	
ON			
History of ON	103	20.9	
No history of ON	366	74.2	
Unknown	24	4.9	
Severe comorbidities			
Neurological	26		
Ophthalmological	15		
Psychiatric	2		

RRMS: relapsing remitting multiple sclerosis; SPMS: secondary progressive multiple sclerosis; PPMS: primary progressive multiple sclerosis; EDSS: expanded disability status scale (Kurtzke, 1983); ON: optic neuritis

### Materials

The SVCq [7] is a questionnaire that aims to screen for visual complaints and was specially designed for people with Parkinson’s disease, dementia and MS. Participants who wear glasses or contacts were instructed to complete the items as if they were wearing them. In the first part of the questionnaire, patients have the opportunity to spontaneously report any visual complaints they may have. Then, 19 statements regarding their sight are presented. Patients were asked to rate each statement on frequency of occurrence (never/hardly, sometimes, often/always). At the end of the SCVq, people indicated the discomfort in daily life due to the visual complaints on a scale from 0 to 10. For the analysis in the current study, we only used the 19 structured items.

### Procedure

People of the MSCNN received an invitation to complete the questionnaire either during a regular visit to their neurologist (n = 409), or via postal mail (n = 198). The invitation comprised a small information letter and the link to a Qualtrics questionnaire [11]. People could complete the online questionnaire in their own time whenever and wherever suited best, on a self-chosen device. One individual requested to complete the questionnaire on paper. People who did not complete the questionnaire initially, were telephoned once for a reminder. These people were also offered to complete the SCVq right away on the phone with a research assistant. The research assistants were instructed to only read the complaint descriptions and answer options to the respondents. The questionnaires were completed between July 2017 and November 2020. Medical data was obtained from the electronic patients records. All pwMS who were invited to complete the SVCq were asked for consent to use the data of the SVCq and the electronic patient records.

### Statistical analysis

#### Confirmatory factor analysis (CFA)

The CFA was performed in LISREL 8.8 [12] to determine if the one-factor model, the three-factor model and the five-factor model of the SVCq in a healthy population [7] could be replicated in a cohort of pwMS. Because of the ordinal structure of the item answers and the non-normality of the responses, we chose to make use of the diagonally weighted least squares (DWLS) method of estimation. All factor variances were set to 1.0.

The fit of the models was assessed according to the following statistics for goodness of fit: (1) normed chi-square. The normed chi-squares were calculated by dividing the Satorra Bentler chi-square value by the degrees of freedom (*χ*^2^/*df*) of the model. This parameter takes sample size into account. Values between 2.0 and 5.0 were considered acceptable, values below 3.0 indicated a good fit [13, 14]; (2) Root Mean Squared Error of Approximation (RMSEA) with a 90% confidence interval [15]. The RMSEA should not be larger than 0.07 and the upper limit of the confidence interval should not exceed 0.08 for an acceptable fit [13, 16]. (3) Standardized Root Mean Square (SRMS), which varies between 0 and 1. Values of 0.08 or smaller indicated a good model fit [17]. (4) Comparative Fit Index (CFI), with values indicative of a good fit between 0.90 and 0.95 [18]. To statistically compare the fit of the three proposed models, we performed nested chi-square difference tests for ordinal data [19].

### Internal consistency

McDonald’s ω was calculated in SPSS (v26.0) for every factor to assess internal consistency between the items. For the *ocular discomfort* scale, we calculated a Spearman Brown coefficient, since this measure is most appropriate to use for 2-item scales [20]. Values between above 0.70 indicate an acceptable internal consistency [21]. Values over 0.95 may indicate items who do not have any individual added value to the questionnaire [22].

## Results

### Confirmatory factor analysis

In total, 493 pwMS completed the questionnaire. There was no missing data, hence the analysis was performed with all 493 pwMS. The item loadings and fit statistics of the three models from the CFA are shown in Table 2. Values of the RMSEA, SRMR, and CFI were all within the proposed intervals, indicating a good fit for all models. The normed chi-square indicated a good fit of all the models. Nested chi-square difference tests showed that the five-factor model had a significantly better fit compared to both the one-factor model (χ^2^(10) = 42.37, *p* =  < 0.001) and the three-factor model (χ^2^(7) = 1.14, *p* =  < 0.001). The fit of the one- and three-factor models did not differ statistically from each other (χ^2^(3) = 4.42, *p* =  < 0.220). Dividing *diminished visual perception* into three additional factors (creating the five-factor model) was beneficial to the model fit.

**Table 2:** Factor loadings and goodness-of-fit-statistics of the CFA on three models of the SCVq

Item no	Factor loadings		
	1-factor model	3-factor model	5-factor model
2	0.80	0.81	0.83
3	0.83	0.83	0.86
20	0.85	0.86	0.88
5	0.90	0.91	0.93
9	0.78	0.78	0.80
10	0.90	0.90	0.98
11	0.83	0.84	0.90
12	0.86	0.86	0.94
17	0.84	0.85	0.91
18	0.75	0.75	0,79
19	0.94	0.94	1.00
4	0.54	0.58	0.58
6	0.60	0.65	0.65
7	0.84	0.90	0.90
8	0.85	0.92	0.92
13	0.39	0.42	0.42
14	0.51	0.55	0.55
15	0.47	0.61	0.61
16	0.45	0.56	0.56
*Fit statistics*			
χ^2^ (df)^a^	394.30 (152)	357.75 (149)	286.15 (142)
χ^2^/*df*	2.59	2.40	2.02
RMSEA	0.057	0.053	0.045
CI-RMSEA	0.050; 0.064	0.046; 0.060	0.038; 0.053
SRMR	0.071	0.066	0.061
CFI	0.99	0.99	0.99

χ^2^/*df* = normed chi-square, CFI: Comparative Fit Index, CI-RMSEA: 90% confidence interval of RMSEA, RMSEA: Root Mean Squared Error of Approximation, SRMR: Standardized Root Mean Square Residual

^a^Satorra-Bentler Scaled Chi-Square

### Internal consistency

The internal consistency of the one-factor model was good (McDonald’s ω = 0.89). In the three-factor model, internal consistencies of the factors were good for *diminished visual perception* (0.89), moderate for *altered visual perception* (0.66) and poor for *ocular discomfort* (Spearman Brown coefficient: 0.40). When dividing *diminished visual perception* into the three additional factors to create the five-factor model, *diminished-function related* and *diminished-luminance related* showed good internal consistencies (0.81–0.73 respectively). *Diminished-task related* showed a moderate internal consistency between the items (0.70).

## Discussion

The SVCq was developed to screen for visual complaints in people with a neurodegenerative disorder, including MS. In a study regarding the psychometric values of the SVCq a factor analysis was performed in a healthy population [7]. The one-factor model, along with the three-factor model and the five-factor model that were revealed by the factor analysis all showed good psychometric values. The objective of the present study was to investigate the goodness-of-fit of the one-factor model (including all items in one factor), the three-factor model and the five-factor model in a sample of pwMS, using CFA.

The CFA revealed that all three tested models had a good fit in the cohort of pwMS. Though the differences were small, the three-factor model showed a better fit than the one-factor model and an extra division of one of the three factors (*diminished visual perception*) into three separate factors (*function related*, *luminance related*, and *task related*) to create the five-factor model resulted in an even better fit. This indicates that statistically, subdividing the items of the SVCq into three or five subscales has merit. The internal consistencies of the items within the factors slightly decreased with the addition of more factors. This may partly be explained by the inevitable decrease of the number of items per factor, which decreases the internal consistency between the items [23]. This may also account for the lower internal consistencies of the items in the scale of *ocular discomfort* that consists of only two items.

### The SVCq in clinical practice

While the reliability and validity of the questionnaire has only been assessed in a healthy population, it may be helpful to explore how the SVCq may support clinical care for pwMS in the future. Applying the one-factor model (total score on the SVCq) may be useful for a quick and time-efficient measure on the number and severity of the visual complaints pwMS experience. Higher total scores may indicate the need to take further steps in providing care. However, we would not suggest to refer just based on a certain amount of complaints, since the nature of complaints, rather than the number of complaints may be of significance in determining the next steps that should be taken in providing care. Moreover, all complaints may have significant impact on daily life individually. Therefore, we would suggest to make use of the five-factor model in clinical practice, or the three-factor model at least. In case of higher scores on *ocular discomfort*, referral to an ophthalmologist would be specifically appropriate, instead of for example referral to a rehabilitation center for visual problems. Dry eyes and/or painful eyes could be the consequence of a curable eye disorder. Rehabilitation would also not be meaningful when for example dry eyes are caused by the use of certain medications. Second, the other factors, namely the *function-related*, *luminance-related* and *task-related* scales of *diminished visual functioning* and *altered visual functioning* could be considered in tailoring the rehabilitation plan for people with visual complaints, since different complaints might ask for different rehabilitation strategies. Third, we would suggest considering regular assessments of visual complaints in pwMS, since visual complaints may arise anytime along the disease course [6] but may also worsen with disease progression, or as a consequence of an exacerbation. General practitioners, specialists (e.g. ophthalmologists and neurologists), and rehabilitation centers or other clinical institutions could well make use of the SVCq.

### Strengths and limitations

The most prominent strength of this study is the sample size of the cohort. However, it has to be mentioned that a small number of patients (estimated 10–15 pwMS) from the cohort was not asked to complete the questionnaire, due to too severe disability. This could have marginally impacted the fit of the model, but the demographics and disease characteristics of our cohort are representative of a MS-population [24, 25]. Since only pwMS who did complete the SVCq could be inquired for permission to use their data, we do not have demographic and MS-related information about this group. The current sample may even be especially representative for pwMS that may benefit from referral to rehabilitation, since neuro-visual rehabilitation may not be a suitable step for pwMS that are far along the disease course. Moreover, as stated earlier, we only assessed the fit of the proposed models and did not reassess validity or test–retest reliability in our clinical sample. So, while the psychometric values in a healthy population were promising, we cannot claim good reliability or validity in our clinical population at this point. In reassessing the validity and reliability, it is advised to take the changing and unpredictable nature of MS into account. Like the validity and determining reliability in the healthy population, our study can only draw conclusions regarding the Dutch version of the SVCq in a Dutch-speaking population. Validity and reliability should be explored for translations of the SVCq. Another issue that should be pointed out is that we cannot exclude potential mode effects from the methods of data collection. People either completed the questionnaire online by themselves after the invitation (n = 379), or by themselves after a reminder from a research assistant, or directly on the phone with a research assistant (n = 114). We do not know which people completed the questionnaire on the phone. Since people could complete the questionnaire by themselves, we do not know if any help from family or other caretakers was provided during the completion of the SVCq.

## Conclusions

The high prevalence and large variability of visual complaints in pwMS and its impact on daily life indicates that it is of clinical importance to bring these complaints to our attention. Using the three different models of the SVCq that were confirmed in a cohort of pwMS may serve different purposes in clinical care and rehabilitation. In future use, the total score of the SVCq (one-factor model) may provide a quick overview of the presence and severity of visual complaints. Subdividing *diminished visual perception* into three separate factors, creating the five-factor model, increases fit and may ameliorate further guidance in determining the next steps in organizing the appropriate care for pwMS who experience visual complaints.

## Data Availability

The datasets used and/or analyzed during the current study are available from the corresponding author on reasonable request.

## Abbreviations

MS: Multiple sclerosis
pwMS: People with multiple sclerosis
SVCq: Screening Visual Complaints questionnaire
NEI-VFQ: National Eye Institute-Visual function questionnaire
MSVQ: MS-specific vision questionnaire
ICC: Intraclass correlation coefficient
CFA: Confirmatory factor analysis
MSCNN: MS Centrum Noord Nederland
RRMS: Relapsing remitting multiple sclerosis
SPMS: Secondary progressive multiple sclerosis
PPMS: Primary progressive multiple sclerosis
EDSS: Extended Disability Status Scale
DWLS: Diagonally Weighted Least Squares
RMSEA: Root Mean Squared Error of Approximation
SRMS: Standardized Root Mean Square
CFI: Comparative Fit Index
